# Measuring Sulforaphane and Its Metabolites in Human Plasma: A High Throughput Method

**DOI:** 10.3390/molecules25040829

**Published:** 2020-02-13

**Authors:** Annie Langston-Cox, Dovile Anderson, Darren J. Creek, Kirsten Palmer, Euan M. Wallace, Sarah A. Marshall

**Affiliations:** 1 Department of Obstetrics and Gynaecology, Monash University, Monash Medical Centre, 246 Clayton Road, Clayton, VIC 3168, Australia; annie.cox@monash.edu (A.L.-C.); kirsten.palmer@monash.edu (K.P.); Sarah.marshall@monash.edu (S.A.M.); 2 Drug Delivery, Disposition and Dynamics, Monash Institute of Pharmaceutical Sciences, Monash University, Parkville, VIC 3052, Australia; dovile.anderson@monash.edu (D.A.); Darren.creek@monash.edu (D.J.C.)

**Keywords:** sulforaphane, Liquid Chromatography–Mass Spectrometry, pharmacokinetic

## Abstract

(1) Background: There is increasing understanding of the potential health benefits of cruciferous vegetables. In particular sulforaphane (SFN), found in broccoli, and its metabolites sulforaphane-glutathione (SFN-GSH), sulforaphane-cysteine (SFN-Cys), sulforaphane cysteine-glycine (SFN-CG) and sulforaphane-N-acetyl-cysteine (SFN-NAC) have potent antioxidant effects that may offer therapeutic value. Clinical investigation of sulforaphane as a therapeutic antioxidant requires a sensitive and high throughput process for quantification of sulforaphane and metabolites; (2) Methods: We collected plasma samples from healthy human volunteers before and for eight hours after consumption of a commercial broccoli extract supplement rich in sulforaphane. A rapid and sensitive method for quantification of sulforaphane and its metabolites in human plasma using Liquid Chromatography–Mass Spectrometry (LC–MS) has been developed; (3) Results: The LC–MS analytical method was validated at concentrations ranging between 3.9 nM and 1000 nM for SFN-GSH, SFN-CG, SFN-Cys and SFN-NAC and between 7.8 nM and 1000 nM in human plasma for SFN. The method displayed good accuracy (1.85%–14.8% bias) and reproducibility (below 9.53 %RSD) including low concentrations 3.9 nM and 7.8 nM. Four SFN metabolites quantitation was achieved using external standard calibration and in SFN quantitation, SFN-d*_8_* internal standardization was used. The reported method can accurately quantify sulforaphane and its metabolites at low concentrations in plasma; (4) Conclusions: We have established a time- and cost-efficient method of measuring sulforaphane and its metabolites in human plasma suitable for high throughput application to clinical trials.

## 1. Introduction

The past decade has seen increasing interest in the potential health and medicinal benefits of naturally occurring antioxidants. Specifically, the health benefits of cruciferous vegetables have been identified by epidemiological studies [1,2,3], stimulating the identification of the likely active antioxidant components of these food groups. For example, resveratrol [4,5,6], vitamins C [7] and E [8], and selenium [9] have all been studied for their antioxidant therapeutic potential. Sulforaphane is another such plant-derived antioxidant that offers promise as a safe and clinically effective agent [10,11,12,13,14,15].

**Figure 1:**
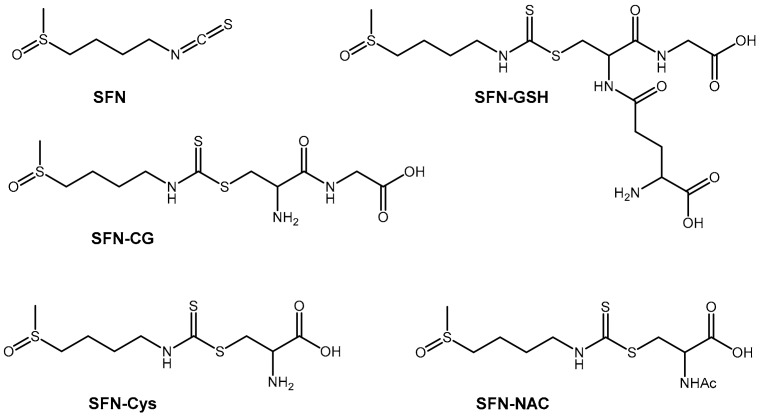
Molecular structures of sulforaphane and its metabolites: Sulforaphane (SFN) sulforaphane-glutathione (SFN-GSH), sulforaphane-cysteine (SFN-Cys), sulforaphane cysteine-glycine (SFN-CG) and sulforaphane-N-acetyl-cysteine (SFN-NAC).

Glucoraphanin is an isothiocyanate found in cruciferous vegetables, particularly young broccoli (sprout and seeds) [16]. Glucoraphanin is converted into the active antioxidant, sulforaphane, in the gastrointestinal tract by myrosinase-catalyzed hydrolysis [16,17]. Sulforaphane is further metabolized into a number of other active metabolites (Figure 1). Sulforaphane and its metabolites are gene transcription activators that release bound nuclear factor erythroid factor 2 (NFE2L2) in the cell cytosol, allowing its nuclear translocation [13,18,19,20,21]. In the nucleus, NFE2L2 induces transcription of a cassette of cellular “safeguarding” genes in the antioxidant response element (ARE) of cellular DNA [22]. This leads to the translation of several antioxidant enzymes that then undergo redox reactions to reduce damaging oxygen free radicals [23].

Initial approaches to the measurement of sulforaphane and its metabolites in biological samples were limited in sensitivity and were unable to accurately quantify levels of metabolites in humans [24,25]. More recently, an approach offering increased sensitivity was described using liquid chromatography–mass spectrometry (LC–MS) [21]. This allowed quantification of sulforaphane and its metabolites at lower concentrations in both plasma and urine [26]. However, it also required extensive plasma clean-up during sample preparation, limiting the number of samples that could be handled at any given time. Given the relative fast thermal degradation of sulforaphane and its conjugates in human plasma at room temperature, with a reported half-life at 24 °C of between 6.16 and 0.49 h [27,28,29] and problems associated with sample degradation during lengthy plasma clean-up, a more rapid sample preparation methodology ahead of LC–MS would both allow processing of high numbers of samples, as would be required in large scale clinical trials, and may improve measurement accuracy. To that end, here we present a modified, cheaper, and high throughput method for the measurement of sulforaphane and its metabolites (Figure 1) using LC–MS.

## 2. Results

### 2.1. Method Development

Method development was based on chromatographic separation and peak shape using pre-consumption (blank) plasma spiked with standards SFN-Cys, SFN-GSH, SFN, SFN-NAC and SFN-CG. Analyte-specific transitions settings (precursor *m*/*z*, Q1 pre-rod bias voltage, product m/z, collision energy and Q3 pre-rod bias voltage) were optimized by a LabSolutions software (Shimadzu, Kyoto, Japan, 2019) automated protocol from flow injection analysis of mixed pure standards at 1 µM concentration. HESI source parameters were optimized manually using flow injection mode.

**Figure 2:**
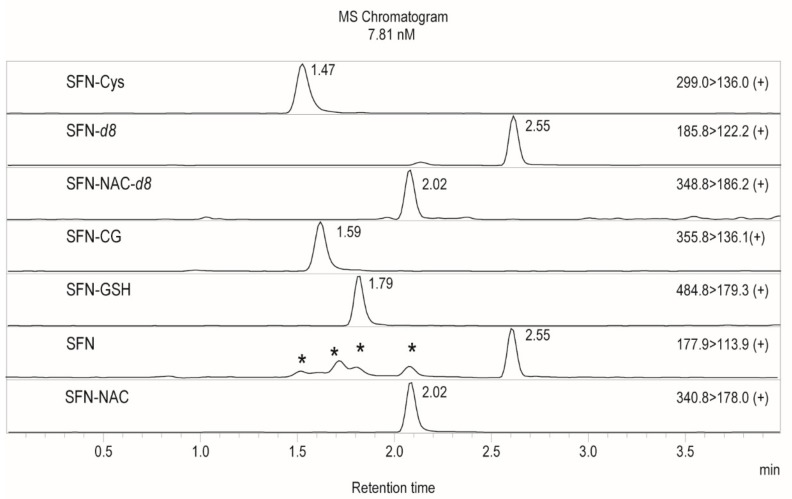
Extracted ion chromatograms of 7.8 nM spiked extracted plasma. SFN and SFN-CG peaks were produced due to in-source fragmentation are marked with arrows. Transitions from the top to the bottom: SFN-Cys, SFN-*d_8_*, SFN-NAC-*d_8_*, SFN-CG, SFN-GSH, SFN, and SFN-NAC. Peaks produced by in source ionization marked with “ * ”.

Baseline peak separation of all analytes was achieved using aqueous reverse phase chromatography. As shown in Figure 2, in-source fragmentation of SFN conjugates was observed which resulted in SFN peaks (transition 177.8 > 113.9 *m*/*z*) being produced at the retention times of SFN-Cys (1.47 min), SFN-CG (1.59 min), SFN-NAC (2.02 min) and SFN-GSH (1.79 min). Similarly, SFN-GSH breakdown in source produced an additional SFN-CG peak at 1.79 min. Thus, good peak separation was important to avoid signal contamination. In-source fragmentation was observed only in plasma samples especially in higher spike concentrations and not in standards prepared in water, suggesting that fragmentation is induced by matrix components of plasma. Changing ESI source parameters, such as voltage or interface temperature, reduced some, but not all, in-source fragmentation. Therefore, a calibration curve for quantitation was constructed in matched matrix–extracted plasma. The observed in-source fragmentation was reproducible and did not impede method accuracy, linearity or precision.

### 2.2. Sample Preparation

Previous LC–MS techniques require plasma sample preparation involving protein precipitation using cold methanol ethyl acetate [30] or trifluoroacetic acid [26,29,31,32]. Further sample clean-up using solid phase extraction techniques (SPE) is often employed [26,30,32,33]. As organic solvents lack extensive buffering capacity and given the sensitive nature of SFN and SFN conjugates, we aimed to eliminate the SPE step to prevent room temperature exposure and unnecessary sample handling, thereby minimizing sample degradation. We used methanolic precipitation of proteins as previously described [34,35] but modified the procedure to accommodate a smaller volume of plasma (25 µL). After extraction methanol was evaporated and sample resolubilized in the same volume of 0.1% formic acid solution to accommodate reversed phase chromatography gradient starting conditions. Attempts to inject methanolic extract resulted in poor peak shape of early eluting peaks and could not be improved by dilution or changing the gradient. Samples, standards, solvents, microcentrifuge tubes and vials were kept on ice throughout the process to minimize degradation of target compounds. Reduction of required plasma amount for analysis is desired in clinical setting, as collected plasma can be aliquoted and used for other tests or diagnostics.

During method validation it has become evident that SFN undergo degradation if exposed to higher than 4 °C temperature even for short periods of time. Also, loss of internal standard SFN-*d_8_* signal in time upon each injection was observed in couple of instances suggesting that perhaps in some batches of plasma SFN degradation is faster. High degree of degradation results in low SFN measurement accuracy. SFN is known to undergo thermal degradation at temperatures above −20 °C [36]. Short-term solution stability of SFN can be increased below pH 3–4. However, exposure to temperatures warmer than 4 °C will accelerate decomposition at acidic conditions. We were able to validate the method and show that if SFN stability is sufficient, the method is accurate and sensitive. However, SFN accuracy will depend on how the samples are handled. It is crucial to keep all samples and standards on ice, use chilled solvents, tubes and vials throughout sample preparation and ensure autosampler temperature has reached 4 °C before samples are loaded. If SFN stability is not ensured, spiking SFN-*d_8_* internal standard will not be able to account for large losses and low accuracy will be obtained.

### 2.3. Accuracy and Linearity

As shown in Table 1, −11.8%–14.8% % bias was observed within linear range 3.9–1000 nM for SFN-GSH, SFN-Cys, SFN-NAC and SFN-CG and within 7.8 nM–1000 nM for SFN. Good accuracy for SFN was achieved by spiking 60 nM SFN-*d_8_* internal standard into extraction solvent (corresponding to 300 nM plasma concentration) and using area ratio in calibration curve generation of SFN. Linear fit with 1/A^2^ weighting factor was used for all target compounds. Good fit to this model was observed as represented by correlation coefficient (R^2^ > 0.99).

### 2.4. Limit of Quantification

For SFN-GSH, SFN-Cys, SFN-NAC and SFN-CG LOQ was 3.9 nM and for SFN–7.8 nM (Table 2).

### 2.5. Precision

Repeatability was below 2% RSD for all target compounds at 200 nM QC level (n = 6) and below 8 %RSD at low concentrations: 3.9 nM and 11.7 nM levels (n = 4) for SFN-GSH, SFN-CG, SFN-Cys and SFN-NAC. For SFN repeatability was 8.61% at 7.8 nM (n = 4). Intermediate precision determined at 200 nM level on 3 different days was below 5.73% RSD for all analytes and 11.9% RSD for SFN (Table 3).

### 2.6. Recovery

High recoveries of all analytes were observed with % difference below 19.61% (Table 3).

### 2.7. Sample Stability in Autosampler

Samples were stable for at least 12 h when kept at 4 °C in the autosampler, as determined from repeated injection of calibration curve dilutions after 12 h. The two calibration curves were nearly superimposable.

### 2.8. Matrix Effects

SFN-GSH, SFN-CG, SFN-Cys and SFN-NAC displayed acceptable matrix effects (between 2.63% and 29.1% difference in peak areas) and did not affect measurement accuracy. SFN suffered from ion suppression, with an 81%–86% decrease in peak area compared to the samples in water. Such large signal suppression resulted in reduced accuracy even when using a matched matrix to prepare calibration curve solutions. Attempts to chromatographically separate the interfering plasma components from the SFN peak were unsuccessful. We have shown that matrix effects in individual plasma samples for SFN were similar (% difference between −78.5% and −88.4% comparing plasma to water), but the accuracy between individual plasma samples was more varied between −23 and 50 %bias purely due to matrix effects. To account for signal suppression and improve accuracy of SFN, 60 nM SFN-*d_8_* was spiked into extraction solvent. Signal ratio SFN/SFN-*d_8_* was used to construct calibration curve which greatly improved the accuracy and method could be validated.

### 2.9. Application of Study Method to Human Samples

**Figure 3:**
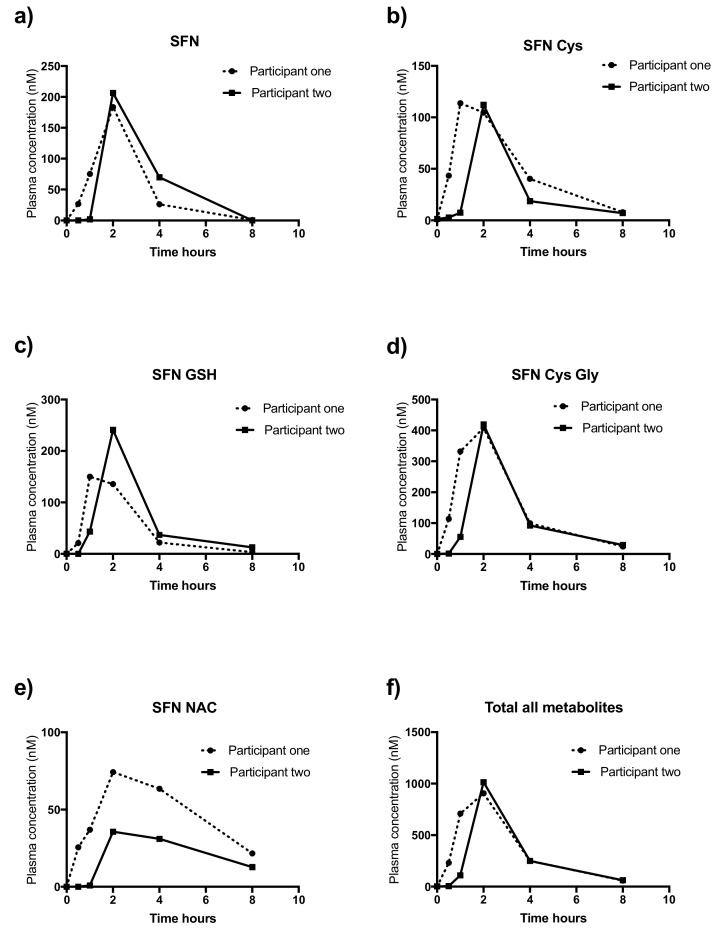
Pharmacokinetic profiles of SFN and metabolites in plasma taken from participant one (dotted line) and participant two (solid line) over 8 h. Metabolites are: (**a**) SFN, (**b**) SFN-Cys, (**c**) SFN-GSH, (**d**) SFN-CG, (**e**) SFN-NAC and (**f**) Total all metabolites (combined value of all metabolites). Y-axis represents measured concentration in ng/ML.

Pharmacokinetic profiles of each metabolite are outlined in Figure 3 for participant one (dotted line) and participant two (solid line) for participant two. As outlined in Table 4, the two participants had similar AUC values for SFN (P1: 424.9 and P2: 520.8), SFN-Cys-Gly (P1: 1264 and P2: 1007) but not for SFN-Cys (P1: 401 and P2: 245.5), SFN GSH (P1: 400 and P2: 530.3), SFN NAC (P1: 385.6 and P2: 172.5) and combined value. (P1: 2876 and P2: 2476). Mean peak value were largely similar; SFN (P1: 183.5 and 206.5) SFN-Cys (P1: 113.8 and P2: 112.2) SFN-GSH (P1: 150.1 and P2: 240.8), SFN-Cys-Gly (P1: 408 and P2: 419.2), SFN NAC (P1: 74.3 and P2: 35.6) and the combined value (P1: 906.2 and P2: 1014).

## 3. Discussion

A simplified methodology to allow high-throughput LC–MS analysis of plasma samples for the measurement of sulforaphane and its metabolites is described. Analysis time is greatly reduced by employing fast chromatography and simple plasma extraction procedure. These methodological simplifications better allow the use of LC–MS to process the large number of samples that are likely to be required in large clinical trials.

A number of sensitive LC–MS methodologies for quantification of SFN and metabolites in various biological samples have been reported [26,29,30,31,32,33,34,35,37,38]. However, few of these methods are suitable for use in human plasma [26,31,34]. All reported methods use triple quadrupole mass spectrometry coupled to High Performance Liquid Chromatography (HPLC) and stable-isotope-labelled internal standard (SIL IS) quantification, SFN-*d_8_* and SFN-NAC-*d_8_* and, with the exception of the method described by Janobi et al. [31], used Butyl-NAC as an internal standard. Early quantification of sulforaphane and its metabolites in human samples was established through cyclocondensation of all metabolites into a single compound rather than independent assessments of each metabolite [25,39,40]. The accuracy of this approach was limited by variation in the efficiency of the cyclocondensation reaction in combining all metabolites ahead of analysis [41,42,43]. To resolve this limitation, methods were established that allowed the quantification of each metabolite with isotope-dilution tandem mass spectrometry [44]. However, even this approach had some limitations. First, reproducibility was limited with inter-assay coefficients of variation as high as 10%, compromising its application to clinical pharmacokinetic studies [44]. The sensitivity of the method was also insufficient to detect the low levels of some of the metabolites in plasma [45]. Finally, reported methodologies included lengthy sample preparation prior to analysis exposing already unstable metabolites to freeze–thaw cycles [26].

Careful selection of SIL IS and optimizing standard concentrations both reduced inter-assay variation and improved sensitivity [26,34,46]. However, these techniques still relied on multiple expensive internal standards introducing costs that would compromise feasibility in the setting of large clinical trials where frequent analysis of multiple samples must be undertaken. Even in the absence of financial concern, not all SIL standards are commercially available; SFN-CysGly, SFN-SGH and SFN-Cys, and so must be synthesized from SFN-*d_8_* in house [34]. Use of an SIL IS method can limit the sensitivity of the analysis by suppressing analyte signal at low concentration, thereby increasing the limit of detection [37].

In our hands, deuterated internal standards SFN-*d_8_* and SFN-NAC-*d_8_* produced remarkably lower signal intensities when spiked at the same concentrations as their non-deuterated versions. Low signals introduced inaccuracies and caused SFN-*d_8_* and SFN-NAC-*d_8_* to fail in the method validation step. We attempted to develop a sensitive and fast SIL-free LC–MS method. SFN conjugates could be quantified successfully using external standard quantification. However, for SFN, this approach proved challenging due to observed matrix effects and possible SFN degradation which resulted in reduced accuracy. This limitation was overcome by spiking extraction solvent with freshly prepared SFN-*d_8_*. We also optimized methods for quantifying SFN metabolites by simplifying plasma preparation. We shortened the run time to eight minutes and used external standard calibration to quantify metabolites. We then confirmed this quantification method in human plasma after consumption of commercial sulforaphane preparation.

The mean peak of combined metabolites from our study (0.9 and 1 μM) using 120 mg of broccoli seed extract (~32 mg of SFN) was similar to work by Fahey et al. who investigated the pharmacokinetics of 350 mg of purified broccoli seed powder (mean 1.3 μM ± 0.5 μM) [47], though our dose was almost three-times less. The pharmacokinetic profiles of our study mirrored those of Fahey et al. in that excretion was complete 8 hrs after consumption. Our intervention peaked slightly later (~2hrs), than that of Fahey (~1 hr), likely due to our use of a capsule rather than liquid [47].

This simplified yet sensitive methodology allows high-throughput LC–MS analysis of plasma samples for the measurement of sulforaphane and its metabolites. The preliminary results confirmed method suitability to study sulforaphane supplementation in patients. Our methodological adaptations better allow the use of LC–MS to process the large number of samples that are likely to be required in future dose-finding studies and large clinical trials [48].

## 4. Materials and Methods

### 4.1. Materials

LC–MS-grade acetonitrile was from Burdick and Jackson (Muskegon, MI, USA). LC–MS-grade formic acid (Optima^TM^) was from Fisher Chemical (Thermo Fisher Scientific, Waltham, MA, USA). Reverse osmosis purified MilliQ water used in LC–MS analysis was from Millipore water purification system (Merck, Darmstadt, Germany). Analytical standards SFN, SFN-*d_8_*, SFN-NAC-*d_8_*, SFN-GSH, SFN-NAC, SFN-Cys were from Toronto Research Chemicals (Toronto Research Chemicals, Toronto, ON, Canada). CysGly (>85%) and pyridine were purchased from Sigma Aldrich (St. Louis, MI, USA). HF Bond Elute^TM^ SPE cartridges C18 (6 mL tube, 500 mg bed) were purchased from Agilent Technologies (Colorado Springs, CO, USA). Myrosinase-activated broccoli sprout extract capsules, Broccomax^TM^, were sourced from Jarrow Formulas (Los Angeles, CA, USA).

### 4.2. Application of Study Methods

A pharmacokinetic study was approved by the Monash Health Ethics Committee (HREC: 17-0000-169A) and conducted in accordance with the National Statement on Ethical Conduct [49]. The two healthy volunteer participants were identified within the community and approached for recruitment. Both participants provided written informed consent before they participated in this study. This research was conducted in accordance with the Declaration of Helsinki and the protocol was approved by an ethics committee (HREC 17-0000-169A). Inclusion criteria were non-pregnant, nulliparous women age 18–35. Exclusion criteria included current use of broccoli sprout extract, pre-existing medical condition (thyroid dysfunction, hepatic disease, renal disease, chronic inflammatory disease, polycystic ovarian syndrome), gastrointestinal disturbance, current infection, smoking, or any current medication (excepting the oral contraceptive pill). The characteristics of the participants are outlined in Table 5.

The two participants fasted from midnight the day prior to commencing the study. Both were admitted to a clinical trial research center and an intravenous cannula placed in their non-dominant arm. Baseline venous blood (5 mL) was collected into ethylenediaminetetraacetic acid (EDTA) tubes immediately placed at 4 °C for 20 min before centrifugation for 20 min at 1200× *g* and 4 °C. Plasma was collected and aliquots immediately stored at −80 °C until analysis.

The participants were then observed consuming four Broccomax^TM^ capsules, each containing 30 mg of broccoli seed extract and a dose of 8 mg of sulforaphane, as per manufacturer certificate of analysis, resulting in a total dose of 32 mg of sulforaphane (120 mg of broccoli seed extract). Otherwise they remained fasted for eight hours following. Further blood samples were collected into EDTA tubes at 30 min, one hour, two hours, four hours and eight hours after ingestion of the capsules and processed as above. For the eight-hour period the participants were monitored for potential side effects, reported or observed.

### 4.3. Chemical Synthesis of SFN-CG

*DL*-SFN-CG was synthesized using a modification of the methods described by Kassahun et al. [38] and Hauder et al. [26]. In our modified method NaOH solution pH 8.0 was replaced with pyridine, a Lewis base. 7.24 mg of CysGly (4 eq, 0.04044 mmol) dissolved in 100 µL of 50% EtOH and 1.8 mg of SFN (1 eq, 0.01015 mmol) dissolved in 100 µL of EtOH were mixed together in an Eppendorf tube. Three drops of pyridine were added using a syringe. Pyridine was used in excess and thus the exact amount was not measured. The reaction mixture was stirred for 5 h in the dark. Reaction progress was monitored qualitatively using LC–MS to check if levels of SFN changed. After 5 h the reaction was stopped using 10 µL 1M HCl. The reaction mixture was then diluted with MilliQ water to a volume of 600 µL. Two subsequent SPE clean-up steps were performed. C18 SPE cartridges were washed with 6 mL 0.1% formic acid in acetonitrile and conditioned with 6 mL of 0.1% formic acid in water. The reaction mixture was loaded and washed with 2 mL 0.1% formic acid in water. The reaction product was eluted using 2 mL 10% acetonitrile 0.1% formic acid solution, collecting 0.5 mL fractions. Two subsequent SPE purifications were used to purify SFN-CG. The first SPE purification filtered out pyridine but did not remove excess of CysGly due to the ethanol content in the crude reaction mixture. After the first SPE purification, fractions 1–5 were collected and concentrated under nitrogen stream at 20 °C. After the second purification the pure product was obtained from fractions 2–5 after drying under vacuum.

In total, 2.1 mg (58% yield) of white solid; HRAM ESI-MS(+): *m*/*z* 356.07617 (100; [M + H], C_11_H_22_O_4_N_3_S_3_, delta ppm −1.474); ^1^H NMR (600 MHz, DMSO-*d_6_*), δ 1.67 (m, 4 H, CH), 2.52 (s, 3 H, CH3), 2.66 and 2.77 (m, 2 H, CH), 3.34 (dd, 1 H, CH), 3.50 (dd, 1 H, CH), 3.60 (t, 3 H, CH_2_ and CH), 3.76 (s, 2 H, CH_2_).)

### 4.4. Spectroscopic Data of Standard

SFN-CG structure and purity were confirmed using high resolution accurate mass (HRAM) and ^1^H NMR analyses. HRAM was performed on QExactive HF mass spectrometer (Thermo Scientific, Waltham, MA, USA). Synthesized powdered SFN-CG was dissolved in water and directly infused using a syringe pump into mass spectrometer at 3 µL/min flowrate. The spectra recorded in positive ion mode. ^1^H NMR spectrum was recorded on Bruker Avance 600 MHz spectrometer (Bruker, Billerica, MA, USA).

### 4.5. Preparation of Stock Solutions

In total, 100 mM stock solutions of SFN-GSH, SFN-Cys, SFN-CG and SFN-NAC were prepared in water with formic 0.1% and stored at −80 °C. SFN solution was prepared as 100 mM in EtOH with 0.1% formic acid. Individual stock solutions were mixed to prepare a 1 mM working solution. This solution was serially diluted with 0.1% formic acid solution to prepare 100 µM, 10 µM and 1 µM and 100 nM working solutions that were then used to spike QC samples and allow generation of calibration curve dilutions. All working solutions were prepared daily and discarded after use.

### 4.6. Preparation of Plasma Samples for LC–MS

Single thawed plasma aliquots were used during method development and analysis. 25 µL of plasma was transferred to Eppendorf tubes with 100 µL of pre-chilled 0.1% formic acid in methanol spiked with 60 nM of SFN-d8 (corresponding to 300 nM plasma concentration), all kept on ice. Samples were quickly vortexed and shaken at 4 °C for 4 min. Samples were centrifuged at 1480× *g*, 4 °C for 10 min. Then, 100 µL of supernatant was transferred to clean Eppendorf tubes and the solvent evaporated at 20 °C under nitrogen stream for 30 min. Samples were resolubilized in 100 µL of 0.1% formic acid in water, quickly vortexed and sonicated in a water bath for 30 min with water bath temperature maintained below 25 °C. The samples were centrifuged at 1480× *g* at 4 °C for 10 min and the supernatant transferred into LC–MS vials. The samples were placed into LC–MS compartment (4 °C) and analyzed without delay.

### 4.7. Liquid Chromatography–Mass Spectrometry

Liquid chromatography–mass spectrometry measurements were performed using triple-quadruple mass spectrometer Shimadzu LC–MS8050 (Shimadzu, Kyoto, Japan)coupled with UHPLC system Nexera X2. Electrospray ionization in positive acquisition mode and multiple reaction monitoring mode (MRM) was used. Electrospray source parameters were set as follows: interface voltage 4.0 kV, interface temperature 300 °C, desolvation temperature 250 °C, heat block temperature 300 °C, nebulizing gas, heating gas and drying gas flow 3.0, 8.0 and 10.0 L/min respectively, CID gas pressure 270 kPa. Chromatographic separation was performed on an Hypersil Gold C18aq column (1.9 µm particles, 150 × 2.1 mm, Thermo Scientific) equipped with a guard column (C_18_, 4.0 × 2.0 mm). The mobile phase consisted of 0.1% formic acid in water (A) and in acetonitrile (B). Injection volume was 2 µL. The gradient started at 10% B at time 0 and increased to 99% B by 3.5 min, kept at 99% B until 4.2 min and then returned to 10% B at 4.3 min and kept at 10% B until 8 min.

### 4.8. Method Validation

Method validation criteria were adopted from FDA recommendations on chromatographic bioanalytical method validation [50]. The method was validated using QC samples prepared by spiking pooled pre-consumption time point plasma (blank plasma) from multiple patients at LLOQ, low, medium and high concentrations 3.9 nM or 7.8 nM, 11.7 nM, 200 nM and 1000 nM. Calibration curve solutions were prepared in the matched matrix keeping plasma components concentration the same in all dilutions. The calibration curve covered the range 3.9 nM–1000 nM and was generated by serial dilution of extracted spiked sample (1000 nM spike of all standards) with extracted unspiked sample. Accuracy is the closeness of the measurement to the true value. Accuracy was determined by measuring QC samples at four concentration levels 3.9 nM or 7.8 nM, 11.7 nM, 200 nM and 1000 nM and is expressed as % bias. Recovery is extraction efficiency of sample preparation procedure and is expressed as % of the nominal concentration value. Recovery was determined comparing samples spiked before and after extraction at concentrations levels 40 nM, 200 nM and 1000 nM. Spiking 80% of the nominal concentration values after extraction accounts for 20% of sample volume loss during supernatant transfer step (100 uL is transferred from total 125 uL of sample). Matrix effects, signal suppression or enhancement from coeluting plasma components were assessed by analysing spiked extracted plasma and spiked water. % difference was calculated between measured values for each analyte at three different concentration levels 40 nM, 200 nM and 1000 nM and between extracted individual blank plasma at 200 nM spike. Autosampler stability was assessed by reanalyzing the same calibration curve samples after 12 h in the autosampler. The limit of quantification (LOQ) is the lowest point of calibration curve with accuracy between 80% and 120%. Intraday precision (repeatability) determined from 6 or 4 consecutive injections of four QC levels −3.9 nM or 7.8 nM (LOQ), 11.7 nM (low QC), 200 nM (medium QC) and 1000 nM (high QC). Intermediate precision was inferred from 200 nM QC sample analysis on three different days.

### 4.9. Statistical Analysis 

Area under the curve (AUC) and mean peak were determined using GraphPad Prism 7.0 (GraphPad, San Diego, CA, USA). These values were not compared statistically due to low numbers (n = 2).

## Figures and Tables

**Table 1:** Summary of accuracy and linear range for sulforaphane and metabolites.

Target Compound	Accuracy (% bias)					LOQ (nM)	Linear Range (nM)	R^2^
	3.9 nM	7.8 nM	11.7 nM	200 nM	1000 nM			
SFN	-	−2.70	-	−11.8	12.2	7.8	7.8–1000	0.9947
SFN-GSH	−5.70	11.3	2.65	2.6	0.1	3.9	3.9–1000	0.9944
SFN-CG	−2.30	8.4	−3.15	3.4	−0.60	3.9	3.9–1000	0.9991
SFN-Cys	3.55	10.3	2.65	14.8	7.90	3.9	3.9–1000	0.9962
SFN-NAC	1.85	−4.60	−1.85	11.2	3.20	3.9	3.9–5000	0.9981

**Table 2:** Monitored transitions and retention times of all analytes.

Compound	*m*/*z*, Transition	Collision Energy	Retention Time (min)
SFN-Cys	299.00 > 136.00	−11	1.47
SFN-GSH	484.80 > 179.30	−25	1.79
SFN	177.90 > 113.90	−12	2.55
SFN-NAC	340.80 > 178.00	−14	2.02
SFN-*d_8_*	185.80 > 122.20	−10	2.55
SFN-CG	355.80 > 136.10	−12	1.59

**Table 3:** Summary of precision and recovery for sulforaphane and metabolites.

Target Compound	Repeatability (*n* = 6) %RSD					Intermediate Precision (*n* = 3) %RSD	Recovery (% Difference)		
	3.9 nM	7.8 nM	11.7 nM	200 nM	200 nM		40 nM	200 nM	1000 nM
SFN	-	8.61	-	0.554	11.9		10.57	14.1	−1.64
SFN-GSH	9.53	8.18	7.76	1.96	5.73		9.81	19.61	−6.15
SFN-CG	3.68	4.28	3.95	1.19	5.47		15.2	−10.1	0.846
SFN-Cys	4.01	5.95	1.03	1.72	1.85		−5.63	−1.97	−0.26
SFN-NAC	6.66	3.38	3.12	1.25	0.656		−8.92	−4.67	−3.12

**Table 4:** Area under the curve (AUC) and mean peak of Participants one and two.

Metabolite	Participant One		Participant Two	
	AUC	Mean Peak	AUC	Mean Peak
SFN	424.9	183.5	520.8	206.5
SFN Cys	401	113.8	245.5	112.2
SFN-GSH	400	150.1	530.3	240.8
SFN-Cys-Gly	1264	408	1007	419.2
SFN-NAC	385.6	74.3	172.5	35.6
Total all metabolites	2876	906.2	2476	1014

**Table 5:** Summary of participant demographics.

Demographics	Participant One	Participant Two
Age (yrs)	23	20
BMI (m/kg^2^)	24	26
Dietary restrictions	Nil	Nil
Medication	OCP^1^	Nil
Co-morbidities	Nil	Nil

^1^ Oral contraceptive pill.
